# Easy clinical predictor for low BCAA to tyrosine ratio in chronic liver disease patients with hepatocellular carcinoma: Usefulness of ALBI score as nutritional prognostic marker

**DOI:** 10.1002/cam4.3908

**Published:** 2021-05-07

**Authors:** Atsushi Hiraoka, Masaya Kato, Kaori Marui, Taisei Murakami, Kei Onishi, Tomoko Adachi, Junko Matsuoka, Hidetaro Ueki, Takeaki Yoshino, Miho Tsuruta, Toshihiko Aibiki, Tomonari Okudaira, Taira Kuroda, Ryuichiro Iwasaki, Yoshifumi Suga, Hideki Miyata, Tomoyuki Ninomiya, Masashi Hirooka, Masanori Abe, Bunzo Matsuura, Kojiro Michitaka, Yoichi Hiasa

**Affiliations:** ^1^ Gastroenterology Center Ehime Prefectural Central Hospital Matsuyama Japan; ^2^ Department of Gastroenterology and Metabology Ehime University Graduate School of Medicine Ehime Japan

**Keywords:** albumin–bilirubin score, amino acid imbalance, branched‐chain amino acid to tyrosine ratio, hepatocellular carcinoma, modified ALBI grade, nutrition

## Abstract

**Background/Aim:**

Low branched‐chain amino acid (BCAA) to tyrosine ratio (BTR) is known as an indicator of amino acid imbalance. We elucidated usefulness of newly developed albumin–bilirubin (ALBI) score as alternative methods of BTR in patients with naïve hepatocellular carcinoma (HCC) retrospectively.

**Materials/Methods:**

In 842 patients with HCC and without BCAA supplementation (71 years, male 614, Child‐Pugh A:B:C = 689:116:37), relationships among BTR and clinical features were evaluated. Of those, 438 patients, with Milan criteria HCC, treated curatively were divided into the high‐BTR (>4.4) (*n =* 293) and low‐BTR (≤4.4) (*n =* 145) groups. The prognostic value of BTR was evaluated using inverse probability weighting (IPW) with propensity score.

**Results:**

The low‐BTR group showed worse prognosis than the other (3‐, 5‐, 10‐year overall survival rates: 88.9% vs. 86.3%/70.5% vs. 78.1%/38.1% vs. 52.3%, respectively; *p* < 0.001). Multivariate Cox‐hazard analysis adjusted for IPW showed elderly (≥65 years) HR 2.314, *p* = 0.001), female gender (HR 0.422, *p* < 0.001), ECOG PS ≥2 (HR 3.032, *p* = 0.002), low platelet count (HR 1.757, *p* = 0.010), and low BTR (≤4.4) (HR 1.852, *p* = 0.005) to be significant prognostic factors. Both serum albumin level (*r* = 0.370, *p* < 0.001) and ALBI score (*r* = −0.389, *p* < 0.001) showed a significant relationship with BTR. Child‐Pugh class B, modified ALBI grade (mALBI) 2a, and mALBI 2b predictive values for BTR were 3.589, 4.509, and 4.155 (AUC range: 0.735–0.770), respectively, while the predictive value of ALBI score for low‐BTR (≤4.4) was −2.588 (AUC 0.790).

**Conclusion:**

ALBI score −2.588 was a predictor for low‐BTR (≤4.4), which was prognostic factors for early HCC patients, and at least patients with mALBI 2b might have an amino acid imbalance.

## Graphical abstract

This study was conducted to elucidate usefulness of newly developed albumin–bilirubin (ALBI) score as alternative methods of BTR for predicting amino acid imbalance that affects prognosis in naïve hepatocellular carcinoma (HCC) patients. ALBI score −2.588 was a predictor for low‐BTR (≤4.4), which was prognostic factors for early HCC patients, and at least patients with modified ALBI grade 2b (>−2.27) might have an amino acid imbalance.

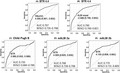

## 1 INTRODUCTION

Hepatocellular carcinoma (HCC) is the most common primary malignancy of the liver and fifth most common malignancy worldwide.^1^ HCC often occurs in patients with chronic liver disease (CLD). The liver is a central organ that controls metabolic nutrition. It is well known that the prognosis of HCC is dependent not only on tumor burden but also hepatic function,^2^, ^3^ as nutritional status generally becomes worse with the progression of liver damage. Notably, decompensated liver cirrhosis (LC) often complicates protein‐energy malnutrition (PEM).^4^ Fischer's ratio has been used for evaluation of plasma free‐amino acid.^1^ A reduced branched‐chain amino acid (BCAA) level is observed during the progression of CLD.^5^ Additionally, BCAA to tyrosine ratio (BTR) is used as an alternative to Fischer's ratio based on a report by Azuma et al.,^6^ which noted BCAA decrease, tyrosine increase, and BTR decline in serum in parallel with the severity of hepatic parenchymal damage. Moreover, the progression of PEM is thought to already exist in patients in early stages of LC.

Suzuki et al. investigated the relationship between yearly change in serum levels of albumin and BTR.^7^ Based on results of a prospective LOTUS trial, in which a long period of oral BCAA granule supplementation improved event‐free survival, serum albumin level, and quality of life (QOL) in decompensated LC patients with an adequate daily food intake,^8^ it is considered that an adequate nutritional intervention should be considered before progression to established PEM. Therefore, establishment of a clinical cut‐off value that can be used as a predictor of early stage of PEM establishment and an easy usable alternative method for predicting PEM in clinical practice are necessary as an indicator of nutritional intervention to improve the prognosis of CLD patients. The present study was conducted to elucidate usefulness of newly developed albumin–bilirubin (ALBI) score as alternative methods of BTR for predicting amino acid imbalance that affects prognosis in naïve hepatocellular carcinoma (HCC) patients.

## 2 MATERIALS AND METHODS

From February 2008 to December 2020, 1270 patients were diagnosed with naïve HCC at our hospital. Their records in an institutional database were analyzed in a retrospective manner.

**FIGURE 1:**
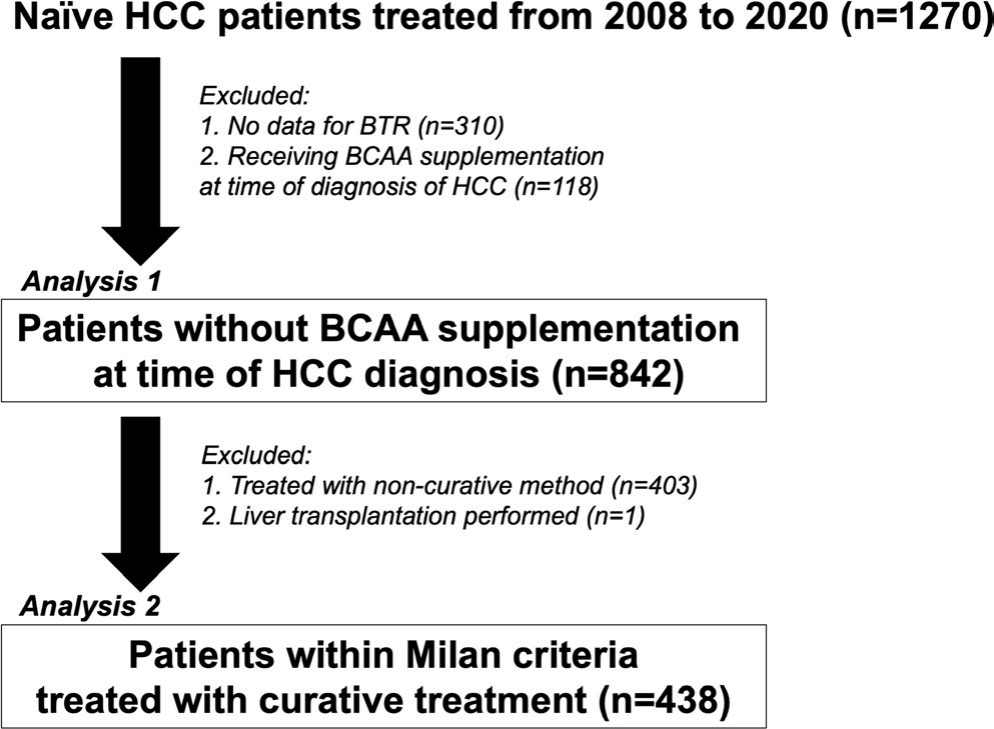
Flowchart of subject enrollment

For Analysis 1, the relationships among BTR and clinical features were investigated. After excluding 310 patients without data for BTR and 118 who were receiving BCAA supplementation at the time of diagnosis of HCC, 842 were enrolled (Figure 1). In Analysis 2, the prognostic value of BTR for survival was evaluated. After excluding 404 patients treated with a non‐curative method, liver transplantation, or best supportive treatment, the prognostic impact of decreasing BTR level was evaluated in 438 patients within the Milan criteria (1 tumor ≤5 cm, or ≤3 tumors with each tumor ≤3 cm)^9^ and given curative treatment (Figure 1). Those 438 subjects were divided into the high‐BTR (>4.4) (n = 293) and low‐BTR (≤4.4) (n = 145) groups.

### 2.1 Basal liver disease

HCC was judged due to hepatitis C virus (HCV) in patients positive for anti‐HCV, while HCC due to hepatitis B virus (HBV) was judged in those positive for the hepatitis B virus surface antigen.

### 2.2 Liver function assessment and BTR determination

For assessment of hepatic reserve function, Child‐Pugh classification^10^ and the HCC treatment algorithm of the Japanese Society of Hepatology^11^ were used. To perform analyses of relationship with BTR and detailed hepatic reserve function, ALBI score^3^, ^12^ and modified ALBI grade (mALBI), with ALBI grade 2 divided into two subgrades (2a and 2b) using an ALBI score of −2.27 as the cut‐off value,^13^ were employed. BCAA, tyrosine, and BTR were measured at time of diagnosis of HCC within 1 month before treatment using a Diacolor‐BTR^®^ kit (before March 2019) and Diacolor‐Liquid BTR^®^ (from April 2019) (TOYOBO CO., LTD., Osaka, Japan), with the lower value of normal BTR range (4.41–10.05)^14^, ^15^ used as the cut‐off for decreasing BTR. The cut‐off value for low‐BTR (≤4.4) was defined based on the results of a previous report.^16^

### 2.3 HCC diagnosis and treatment

HCC was diagnosed based on findings showing an increasing course of alpha‐fetoprotein (AFP), as well as results obtained with dynamic CT^17^ or MRI,^18^, ^19^ contrast enhanced ultrasonography with perflubutane (Sonazoid^®^, Daiichi Sankyo Co., Ltd.),^20^, ^21^ and/or pathological results. For evaluation of tumor progression, tumor node metastasis (TNM) staging, determined based on a previous study of TNM staging for HCC conducted by the Liver Cancer Study Group of Japan (LCSGJ), 6^th^ edition^22^ (TNM‐LCSGJ), was utilized. Surgical resection and radiofrequency ablation (RFA) were each defined as curative treatment.

To analyze the prognostic impact of BTR for overall survival (OS) in Analysis 2, eight clinical factors [gender, age, AFP, Child‐Pugh class, basal hepatic disease etiology, curative treatment method, Eastern Cooperative Oncology Group performance status (ECOG PS), up to 7 criteria score (sum of maximum tumor size and tumor number)] were used for calculating inverse probability weighting (IPW) and propensity score.^23^

This study was based on the Guidelines for Clinical Research issued by the Ministry of Health and Welfare of Japan and all procedures were performed in accordance with the declaration of Helsinki. Informed consent was obtained in the form of opt‐out. Those who rejected were excluded from this study.

### 2.4 Statistical analysis, propensity score calculation, inverse probability weighting

Mean values (standard deviation) are used to express continuous variables. For statistical analyses, Welch's *t*‐test, Student's *t*‐test, Fischer's exact test, Mann‐Whitney's *U*‐test, receiver operator characteristic curve (ROC) analysis, and area under the curve (AUC) were used, with Bonferroni's and Holm's tests employed for multiple comparisons as appropriate. Evaluations of prognosis were done using Cox hazard analysis (stepwise regression method), the Kaplan–Meier method, and a log‐rank test.

For calculating the high‐ and low‐BTR group probabilities (propensity), logistic regression analysis with a set of covariates considered likely to have effects on OS was conducted. IPW was defined as 1/(propensity score) for the high‐BTR group and 1/(1‐propensity score) for the low‐BTR group. The hazard ratio (HR) for OS was determined using IPW‐adjusted Cox hazard analysis.^24^, ^25^

*P* values less than 0.05 were considered to indicate statistical significance. Easy R (EZR), version 1.53 (Saitama Medical Center, Jichi Medical University),^26^ a graphical user interface for R (The R Foundation for Statistical Computing, Vienna, Austria) was used for the statistical analyses.

## 3 RESULTS

### 3.1 Analysis 1

Determination of clinical features in the 842 patients enrolled in Analysis 1 showed median age of 71 ± 10 years, 614 males, ECOG PS values of 0:1:2:3:4:no data in 636:120:42:29:12:3, Child‐Pugh class A:B:C in 689:116:37, CLD etiology showing HCV:HBV:HBV&HCV:alcohol:others:no data in 470:87:10:106:168:1, and TNM‐LCSGJ I:II:III:IVa:IVb in 198:346:176:50:72.

**FIGURE 2:**
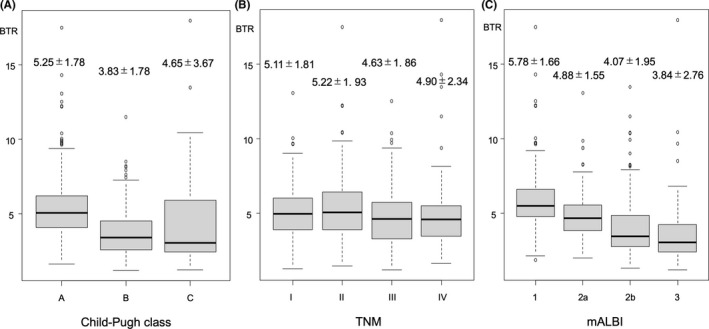
BTR levels based on (A) Child‐Pugh class, (B) TNM‐LCSGJ stage, and (C) mALBI grade. There was a significant difference between Child‐Pugh class A and B (*p* < 0.001, Bonferroni's method). Among the TNM‐LCSGJ stages, there were no significant differences observed except for between TNM‐LCSGJ II and III (*p* = 0.008, Holm's method). There were significant differences among the mALBI grades (*p* < 0.001, Holm's method), except between grades 2b and 3

BTR level was 5.25 ± 1.78 in patients with Child‐Pugh class A, 3.83 ± 1.78 in those with Child‐Pugh class B, and 4.65 ± 3.67 in those with Child‐Pugh class C, with a significant difference found between class A and B (*p* < 0.001, Bonferroni's method) (Figure 2A). Among the TNM‐LCSGJ stages, no significant differences were observed except for between II and III (*p* = 0.008, Holm's method) (Figure 2B). ALBI (mALBI) grade 1 patients showed a BTR level of 5.78 ± 1.66, while it was 4.88 ± 1.55 in mALBI grade 2a, 4.07 ± 1.95 in mALBI grade 2b, and 3.84 ± 2.76 in mALBI grade 3 patients. There were significant differences among the grades (*p* < 0.001, Holm's method), except between grade 2b and 3 (Figure 2C).

### 3.2 Relationships among serum albumin, ALBI score and BTR

**FIGURE 3:**
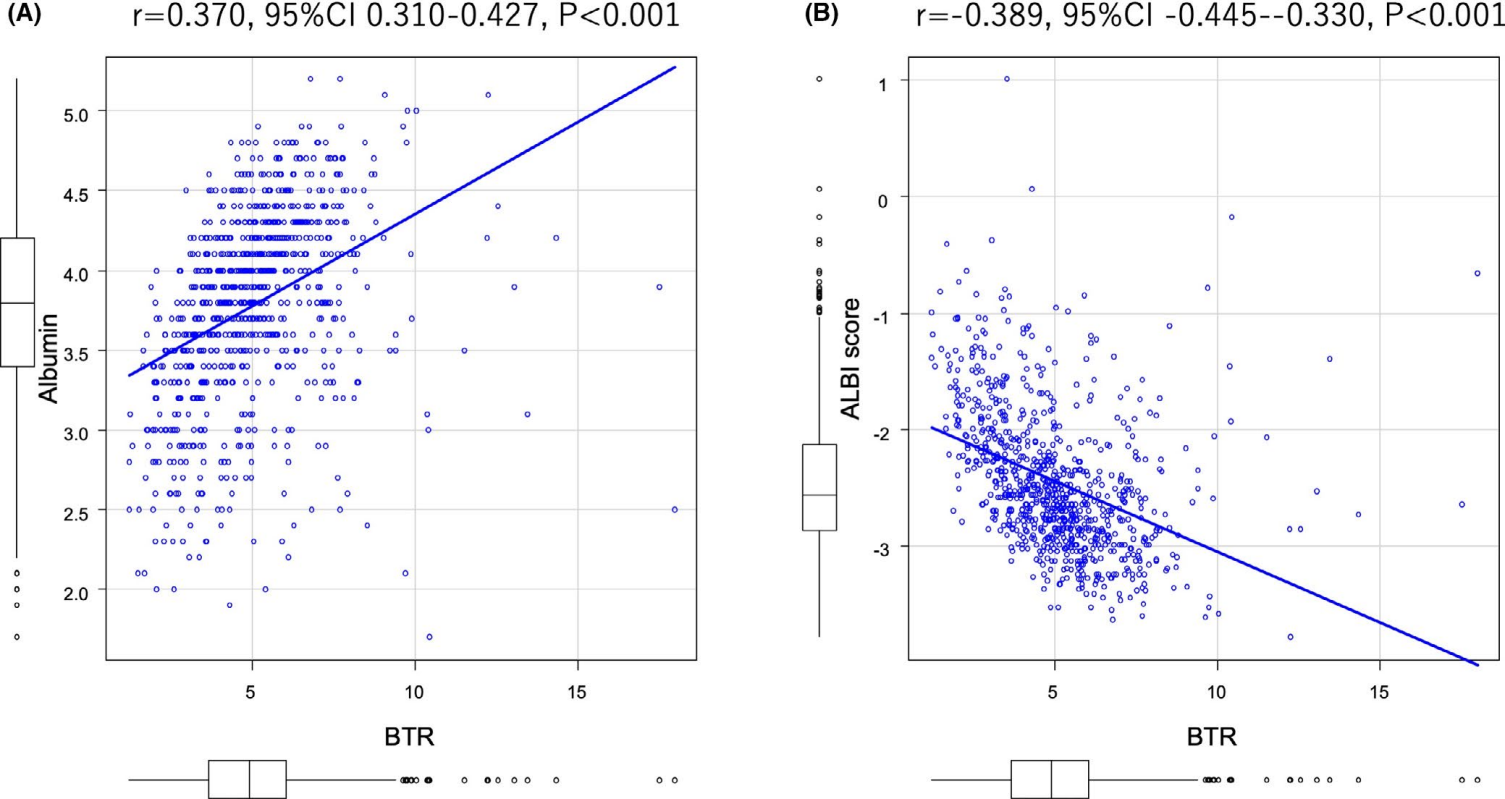
A, Relationship of BTR with serum albumin level. A significantly positive relationship was shown (*r* = 0.370, 95% CI 0.310 to 0.427, *p* < 0.001). B, Relationship of BTR with ALBI score. ALBI score showed a significantly negative relationship with BTR (*r* = −0.389, 95% CI −0.445 to −0.330, *p* < 0.001)

**FIGURE 4:**
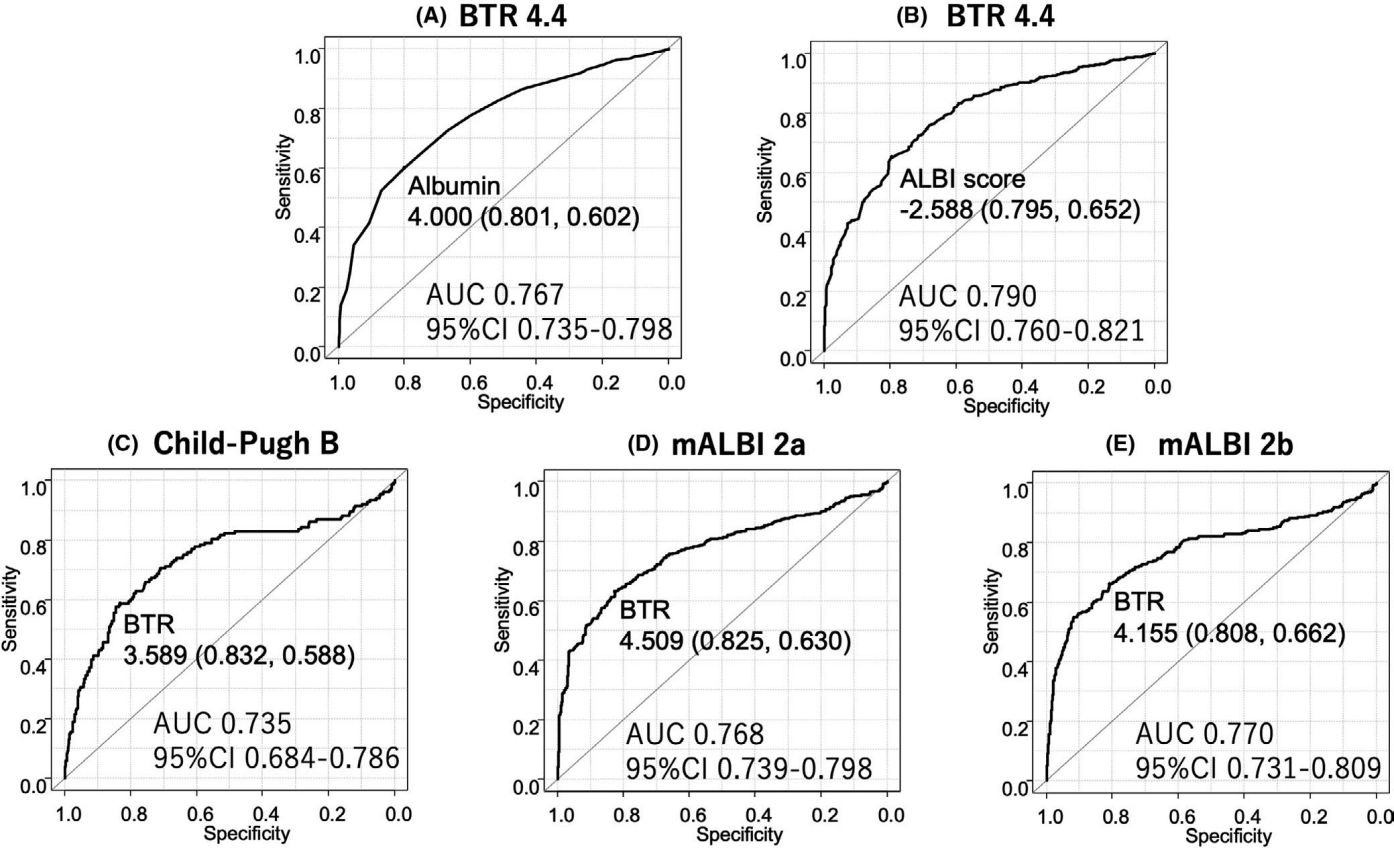
The predictive value of serum albumin for (A) BTR 4.4 was 4.0 g/dL (AUC 0.767, 95% CI 0.735 to 0.798), while that of (B) ALBI was −2.588 (AUC 0.790, 95% CI 0.760 to 0.821), of (C) Child‐Pugh class B was 3.589 (AUC 0.735, 95%CI 0.684 to 0.786), of (D) mALBI grade 2a was 4.509 (AUC 0.768, 95% CI 0.739 to 0.798), and of (E) mALBI grade 2b was 4.155 (AUC 0.770 95% CI 0.731 to 0.809)

Both serum albumin level (*r* = 0.370, 95% CI 0.310 to 0.427, *p* < 0.001) (Figure 3A) and ALBI score (*r* = −0.389, 95% CI −0.445 to −0.330, *p* < 0.001) (Figure 3B) showed a significant relationship with BTR. The predictive value of serum albumin for BTR 4.4 was 4.0 g/dL (AUC 0.767, 95% CI 0.735–0.798) (sensitivity/specificity, 0.801/0.602) (Figure 4A) and that of ALBI score for BTR 4.4 was −2.588 (AUC 0.790, 95% CI 0.760–0.821) (sensitivity/specificity, 0.795/0.652), which was similar to the cut‐off value of mALBI (ALBI) grade 1 (Figure 4B). Child‐Pugh class B, mALBI grade 2a, and mALBI grade 2b predictive values for BTR for 3.589 (AUC 0.735, 95% CI 0.684–0.786) (sensitivity/specificity, 0.832/0.588) (Figure 4C), 4.509 (AUC 0.768, 95% CI 0.739 to 0.798) (sensitivity/specificity, 0.825/0.630) (Figure 4D), and 4.155 (AUC 0.770 95% CI 0.731 to 0.809) (sensitivity/specificity, 0.808/0.662) (Figure 4E), respectively.

There was a significant negative correlation of BCAA level with ALBI score (*r* = −0.234, 95% CI −0.297 to −0.169, *p* < 0.001), while tyrosine level showed a significantly positive correlation (*r* = 0.389, 95% CI 0.330 to 0.445, *p* < 0.001) (Figure S1). Also, the predictive values of BTR for the albumin levels 4.0, 3.9, 3.8, 3.7, 3.6, and 3.5 g/dL were 4.469, 4.308, 3.962, 3.910, 3.547, and 3.593, respectively (AUC range: 0.740–0.755) (Figure S2).

### 3.3 Analysis 2

The 438 patients within the Milan criteria and treated with curative treatment (surgical resection or RFA) were separated based on high‐ and low‐BTR grade, which revealed significant differences for age, gender, basal hepatic disease, platelet count, aspartate transaminase, alanine aminotransferase, total bilirubin, albumin, prothrombin time, ALBI score, BTR, BCAA, tyrosine Child‐Pugh class, tumor number, and treatment. In contrast, frequency of elderly (≥65 years), AFP level, up to 7 criteria score, and TNM‐LCSGJ stage were not significantly different (Table 1).

**TABLE 1:** Clinical features of patients with and without BTR decrease.

	High‐BTR group (n = 293)	Low‐BTR group (n = 145)	*p* value
Age, years	70.0 (9.3)	71.9 (9.2)	*p* = 0.041
≥65 years, n	217, 74.1%	113, 77.9%	*p* = 0.411
Gender, male:female	231:62	85:60	*p* < 0.001
Etiology, HCV:HBV:HBV&HCV:alcohol:other	169:43:6:30:45	105:7:1:13:19	*p* = 0.007
BMI, kg/m^2^	23.6 (3.3)	23.6 (3.5)	*p* = 0.996
ECOG PS, 0:1:2:3:4	242:29:12:9:1	121:19:2:2:1	*p* = 0.308
Platelets, ≥10^4^/µL	15.0 (7.4)	10.7 (4.9)	*p* < 0.001
AST, U/L	41 (27)	56 (28)	*p* < 0.001
ALT, U/L	38 (28)	47 (31)	*p* = 0.002
T‐bilirubin, mg/dL	0.75 (0.53)	1.02 (0.48)	*p* < 0.001
Albumin, g/dL	4.14 (0.45)	3.74 (0.42)	*p* < 0.001
Prothrombin time, %	91.8 (14.1)	83.6 (13.6)	*p* < 0.001
ALBI score	−2.83 (0.39)	−2.38 (0.4)	*p* < 0.001
BTR	6.07 (1.44)	3.37 (0.72)	*p* < 0.001
BCAA	472.3 (112.3)	383.2 (87.5)	*p* < 0.001
Tyrosine	79.93 (19.7)	117.2 (28.9)	*p* < 0.001
Child‐Pugh class, A:B	285:8	125:20	*p* < 0.001
AFP, ng/mL	178.4 (878.9)	140.5 (399.1)	*p* = 0.621
AFP ≥100 ng/mL, n	44, 15.0%	34, 23.4%	*p* = 0.034
Tumor size (maximum), cm	2.2 (1.0)	2.0 (0.8)	*p* = 0.078
Tumor number, single:multiple	247:46	107:38	*p* = 0.027
Up to 7 criteria score	3.39 (1.0)	3.33 (0.9)	*p* = 0.581
TNM‐LCSGJ, I:II:III	125:147:21	55:78:12	*p* = 0.612
Surgical resection:RFA	173:120	109:36	*p* = 0.001
Causes of death (HCC:liver failure:intestinal bleeding:infection:others:unknown)	24 (43.6%):1 (1.8%):1 (1.8%):3 (5.5%):17 (30.9%):9 (16.4%)	17 (39.5%):9 (20.9%):1 (2.3%):2 (4.7%):10 (23.3%):4 (9.3%)	*p = 0.045*
Observation period, years	48.8 (36.0)	47.8 (35.0)	*p* = 0.799
IPW (mean) (SD)	4.32 (2.73)	3.00 (1.51)	*p* < 0.001

Abbreviations: AFP, alpha‐fetoprotein; ALBI score: albumin‐bilirubin score; ALT, alanine aminotransferase; AST, aspartate transaminase; BCAA, branched‐chain amino acid; BMI, body mass index; BTR, branched‐chain amino acid to tyrosine ratio; ECOG PS, Eastern Cooperative Oncology Group performance status; HBV, hepatitis B virus; HCV, hepatitis C virus; IPW, inverse probability weighting; RFA, radio frequency ablation; TNM LCSGJ 6^th^, tumor node metastasis stage by Liver Cancer Study Group of Japan 6th edition.

* Mean values (standard deviation) are shown as numbers, unless otherwise indicated.

### 3.4 Comparisons of recurrence free survival and overall survival using IPW

**FIGURE 5:**
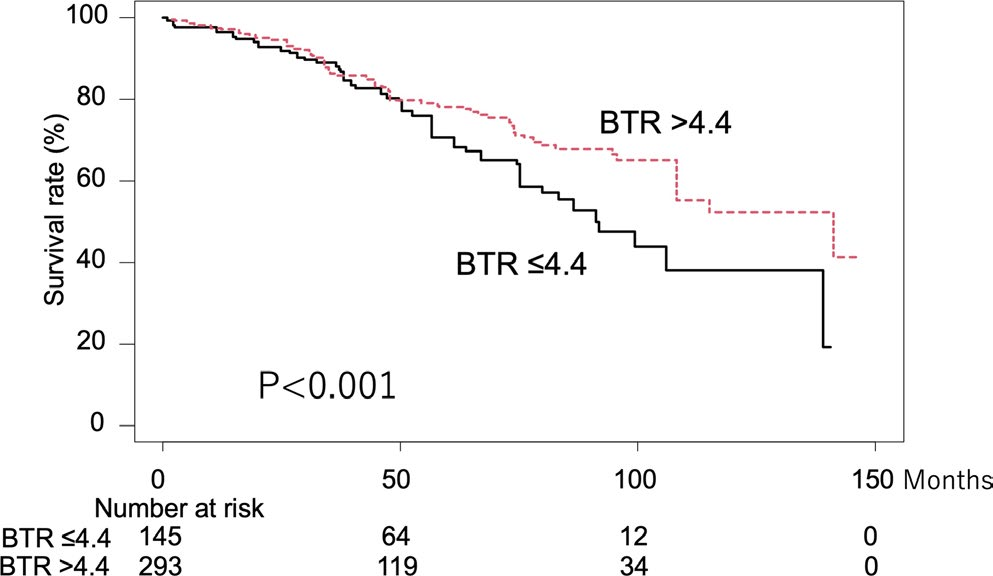
Overall survival (OS) of 438 patients within the Milan criteria treated with curative treatments divided by high‐ and low‐BTR grade, adjusted using IPW. Median survival: 91.3 versus 141.2 months; 3‐, 5‐, and 10‐year OS rates: 88.9% v versus. 86.3%, 70.5% versus 78.1%, and 38.1% versus 52.3%, respectively (*p* < 0.001)

Although there was no significant difference in recurrence‐free survival between the low‐ and high‐groups by log‐rank test results adjusted with IPW (36.6 vs. 38.0 months, *p* = 0.398) (Figure S3), percentage of liver failure as a cause of death were 20.9% in the low‐BTR, while 1.8% in the high‐BTR (Table 1). On the other hand, log‐rank test results adjusted with IPW showed that the low‐BTR group had worse prognosis than the high‐BTR group (median survival time 91.3 vs. 141.2 months; 3‐, 5‐, and 10‐year OS rates: 88.9% vs. 86.3%, 70.5% vs. 78.1%, and 38.1% vs. 52.3%, respectively, *p* < 0.001) (Figure 5). In multivariate analysis, Cox‐hazard (stepwise regression method) analysis with adjustment for IPW [analyzed factors: elderly (≥65 years old), female gender, low body mass index (BMI) (≤20 kg/m^2^), ECOG PS ≥2, HCV infection, low platelet count ( < 10^4^/µL), elevated alanine aminotransferase (≥30 U/L), Child‐Pugh class B, low BTR (≤4.4), elevated AFP (≥100 ng/mL), HCC size ≥2 cm, multiple tumors, surgical resection], the significant prognostic factors were found to be elderly (HR 2.314, *p* = 0.001), female gender (HR 0.422, *p* < 0.001), ECOG PS ≥2 (HR 3.032, *p* = 0.002), low platelet count (HR 1.757, *p* = 0.010), and low BTR (≤4.4) (HR 1.852, *p* = 0.005) (Table 2).

**TABLE 2:** Multivariate analysis of factors related to survival in patients, after adjusting with IPW

	Hazard ratio	95% CI	*p* value
Age (≥65 years)	2.314	1.387–3.861	0.001
Female gender	0.422	0.254–0.700	<0.001
ECOG PS (2 or more)	3.032	1.506–6.103	0.002
Platelet count ( < 10^4^/µL)	1.757	1.143–2.703	0.010
low BTR (≤4.4)	1.852	1.205–2.849	0.005

Abbreviations: BTR, branched‐chain amino acid to tyrosine ratio; ECOG PS, Eastern Cooperative Oncology Group performance status; IPW, inverse probability weighting.

## 4 DISCUSSION

Malnutrition has been shown to be prevalent in LC patients (46%), even those classified as Child‐Pugh class A.^27^, ^28^ Although, a continuing decline in Fischer's ratio as well as BTR, which are factors demonstrating malnutrition, reduces the ability for protein synthesis to occur in the liver,^29^, ^30^ it is a clinical unmet need that there have been few easy usable alternative assessment methods for them. A reduced BCAA level and increased tyrosine level are commonly observed in patients with progression of hepatic function deterioration,^31^ which is termed amino acid imbalance.^32^ Although albumin is one of hepatic function, it alone is not enough to be used as reserve function assessment tool for predicting prognosis and nutritional index. Recent developed ALBI score/ALBI (mALBI) grade has been reported to be able to play roles not only as assessment tools of hepatic function for predicting prognosis ^3^, ^33^, ^34^ but also as nutritional index.^35^ The present results showed that BCAA levels in our cohort had a significantly negative correlation with ALBI score (*r* = −0.234, 95% CI −0.297 to −0.169, *p* < 0.001), while tyrosine level showed a significantly positive correlation (*r* = 0.389, 95% CI 0.330 to 0.445, *p* < 0.001). Based on analysis of the predictive value of BTR for each albumin level (Figure S2) and of ALBI score for BTR 4.4 (−2.588, Figure 4B), the lower limit of the normal range of BTR was located between albumin 4.0 and 3.9 mg/dL, and close to the lower range of ALBI (mALBI) grade 1 (ALBI score −2.60).

PEM was defined based on both protein and energy malnutrition [serum albumin < 3.5 mg/dL, non‐protein respiratory quotient (npRQ) < 0.85]. Tajika et al. reported that PEM was observed in 30% of analyzed LC patients,^4^ while Shiraki et al. noted similar results showing PEM in 27% of their LC patients.^36^ The importance of evaluation of energy metabolism as a predictive prognostic factor in LC patients has been proposed, because a proportional hazards model showed npRQ to be an independent significant factor for survival (relative risk = 0.0003, 95% CI 0.000 to 0.0970).^4^ However, a clinical issue is that assessment of npRQ is difficult for many institutions to perform, and an easy alternative evaluation method or scale for PEM is anticipated. Another report revealed a significantly negative correlation between npRQ and ALBI score (*r* = −0.35, *p* < 0.001), with ALBI score −2.18 the only predictor for npRQ (<0.85) in 109 patients with HCC.^37^ The reported ALBI cut‐off value (−2.18) for npRQ (<0.85) was near the border of mALBI 2a and 2b (ALBI score −2.27). A recent study of the efficacy of the predictive value of mALBI divided into four different grades, a more accurate assessment tool for hepatic function than Child‐Pugh class,^34^ reported that mALBI grade 1 and 2a indicated better prognosis even in unresectable HCC patients treated with molecular targeting agents.^38^, ^39^, ^40^, ^41^, ^42^, ^43^ Decline of BTR (≤4.4) was shown to be a prognostic factor in patients with early HCC treated curatively in the present results (HR 1.852, *p* = 0.005), and the predictive value of ALBI score for low BTR (≤4.4) was −2.588, which was an approximation of cut‐off value of ALBI grade 1, and those of BTR for mALBI grade 2a and 2b were 4.509 (AUC 0.768) and 4.155 (AUC 0.770). Therefore, it is considered that low BTR (≤4.4) may be useful as an approximate nutritional predictor for malnutrition and that at least patients with mALBI 2b might have an amino acid imbalance. When patients show such condition, assessment of nutrition status in detail and starting to consider nutritional intervention might be important for obtaining improving prognosis over a longer period.

BCAA supplementation improves serum albumin level.^44^, ^45^ Muto et al. reported that such supplementation reduced the incidence of negative events (death, HCC development, esophageal varices rupture, progression of hepatic failure) (HR 0.67, 95%CI 0.49–0.93, *p* = 0.015), and hepatic failure (*p* = 0.45, 95%CI 0.23–0.88, *p* = 0.016) (LOTUS trial).^8^ Moreover, muscle abnormality is common in CLD,^46^ and sarcopenia including atrophy and decline of muscle strength are well known as prognostic factors in CLD patients with and without HCC.^47^, ^48^, ^49^, ^50^, ^51^ In a past reports, serum BCAA level showed significant correlations with muscle strength (*r* = 0.189 to 0.238, *p* < 0.01, respectively) and muscle volume (*r* = 0.135 to 0.202, *p* = 0.025 to <0.001) regardless of gender, even in the patients without BCAA administration (*n* = 655), of whom 94.8% were very early CLD such as chronic hepatitis or LC with Child‐Pugh class A.^52^ Therefore, amino acid imbalance ^32^ is thought to already exist in early stages of CLD. Because nutritional intervention was left to the discretion of the attending physicians in the present retrospective analysis, it is very difficult to elucidate clearly the best timing of nutritional intervention with BCAA supplementation after HCC diagnosis. However, adequate nutritional interventions in addition to dietary counseling by a nutrition support team^53^ are needed before conditions worsen to improve the prognosis of CLD patients. Either way, the present results indicate that low BTR level (≤4.4) is an initial timing for considering nutritional intervention (e.g., nutrition management by a registered dietician,^53^ BCAA supplementation) in CLD patients with or without HCC.

The present study has some limitations, including being conducted at a single center and its retrospective nature. A prospective randomized trial that compares outcomes between patients with and without intervention with BCAA supplementation will be needed to obtain a firmer conclusion. Additionally, a more precise cut‐off value for indicating nutritional intervention given to effectively improve prognosis requires a future investigation.

In conclusion, low BTR (≤4.4) might be an effective prognostic factor in CLD patients with early HCC. ALBI score −2.588 was a predictor for low‐BTR (≤4.4), which was prognostic factors for early HCC patients, and at least patients with mALBI grade 2b might have an amino acid imbalance. ALBI score can behave as a useful alternative nutritional marker of BTR.

## CONFLICTS OF INTEREST

Atsushi Hiraoka; lecture fee, Otsuka pharma. All authors are in compliance with the ethical guidelines for authorship and publishing of the *Cancer*.

## AUTHORS’ CONTRIBUTIONS

AH participated in study design, data collection, results analysis, and manuscript writing. MK, KMa, Tm, Ko, TAd, JM, HU, TY, MT, TAi,TO, RI, YS, HM, TN, MA, BM, KMi, and YH participated in data collection and reviewed the manuscript.

## Supporting information

Fig S1

Fig S2

Fig S3

## Data Availability

The datasets generated during and/or analyzed during the current study are not publicly available due to reasons why data are not public.
