# Supplementary figures and images for: Easy clinical predictor for low BCAA to tyrosine ratio in chronic liver disease patients with hepatocellular carcinoma: Usefulness of ALBI score as nutritional prognostic marker
Source: Cancer Med. 2021 May 7;10(11):3584–92. doi: 10.1002/cam4.3908 (PMC8178498; doi:10.1002/cam4.3908)

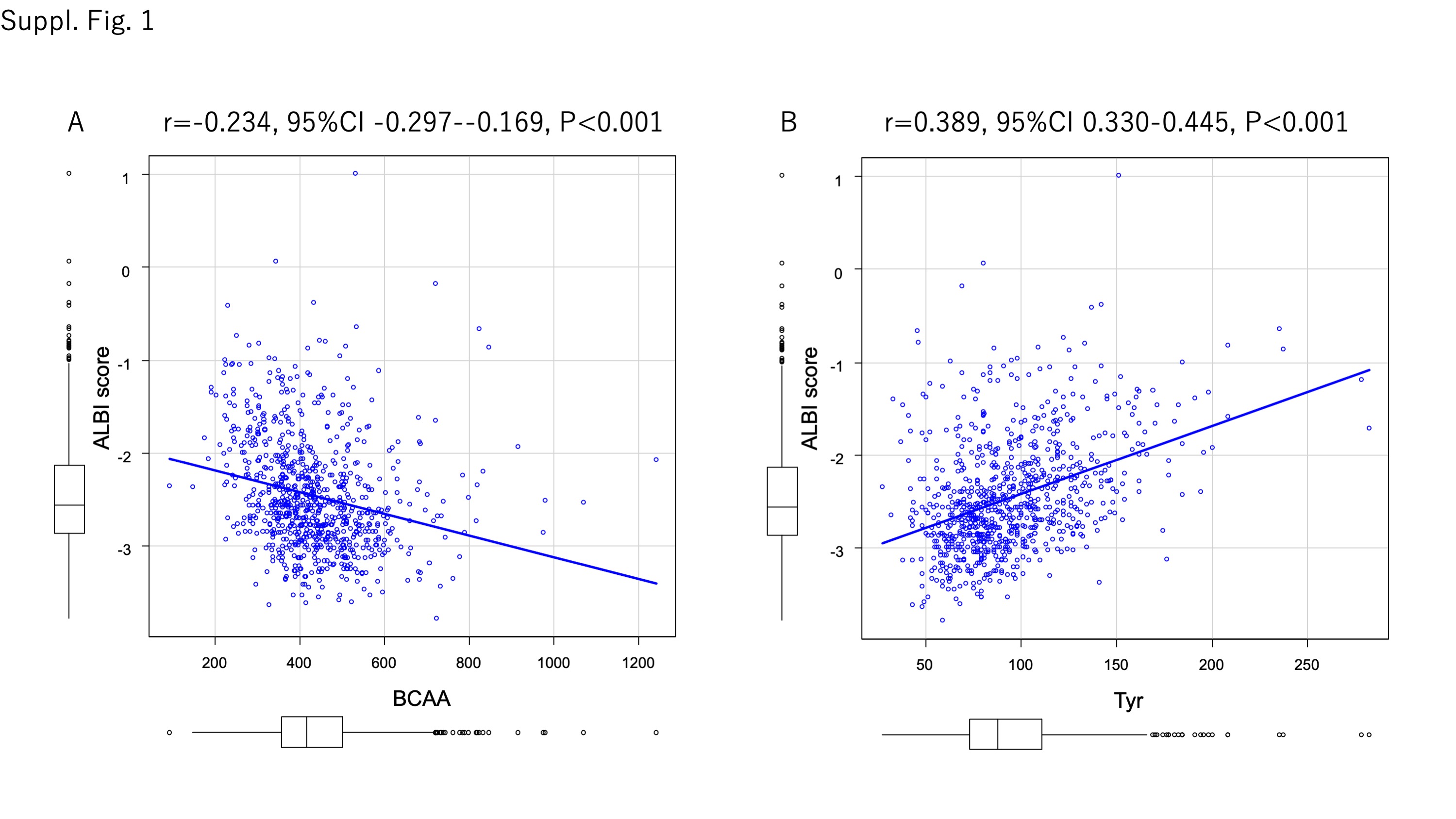

Supplement: Supplementary file 1 — Fig S1 [file CAM4-10-3584-s003.jpeg]

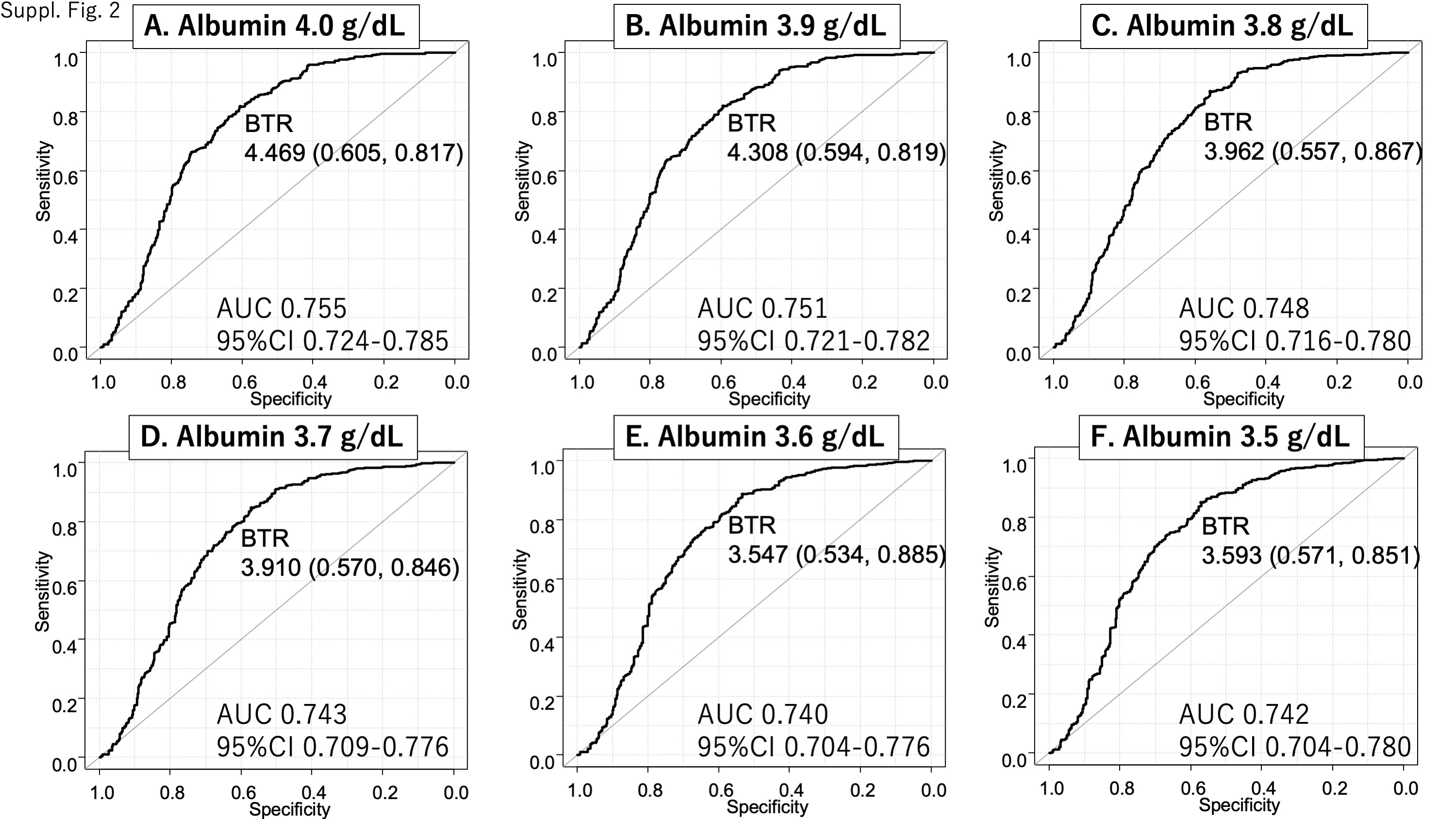

Supplement: Supplementary file 2 — Fig S2 [file CAM4-10-3584-s001.jpeg]

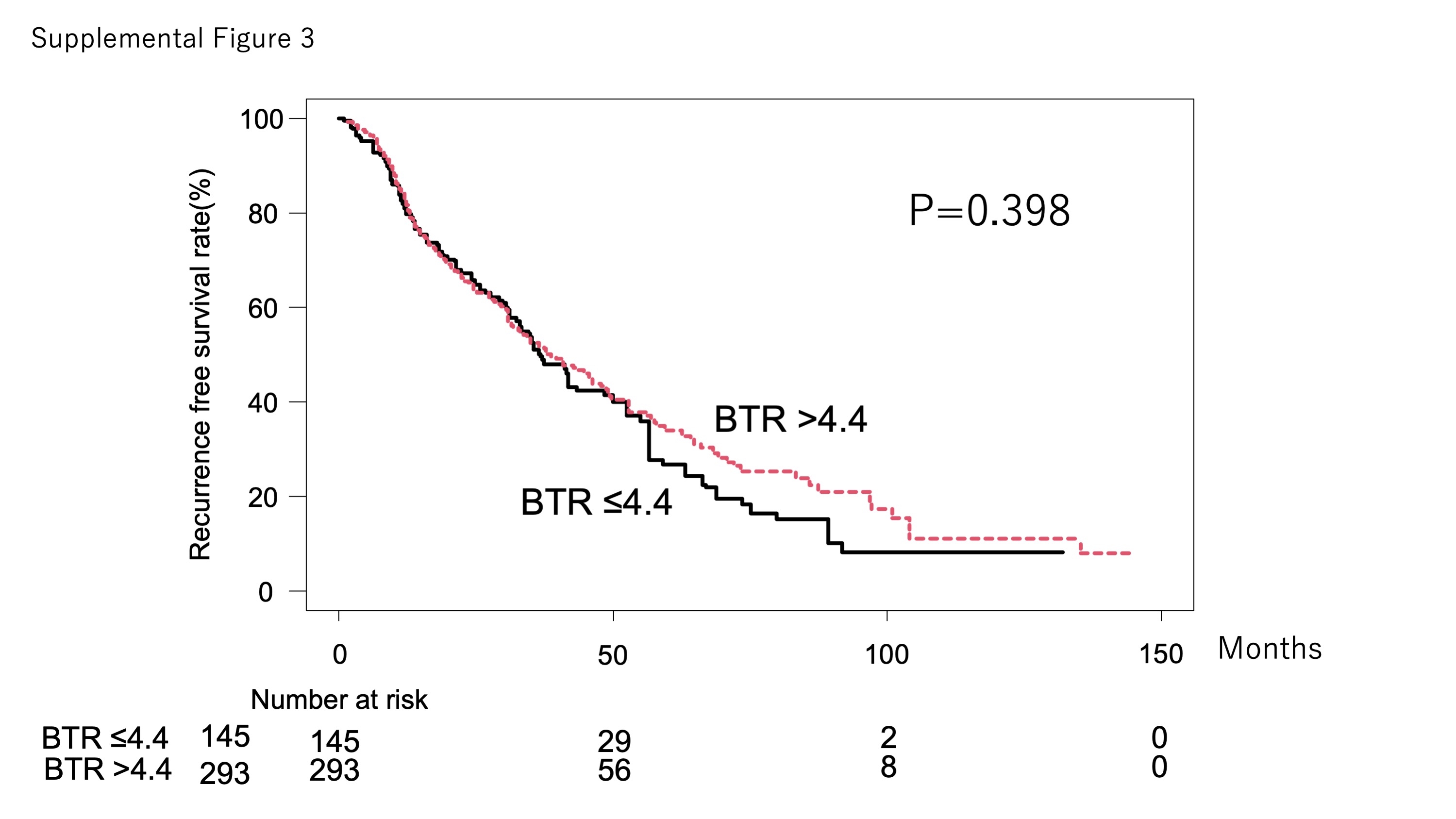

Supplement: Supplementary file 3 — Fig S3 [file CAM4-10-3584-s002.jpeg]
